# Melanie Saville: the end-to-end process needed for a SARS-CoV-2 vaccine

**DOI:** 10.2471/BLT.20.030920

**Published:** 2020-09-01

## Abstract

Melanie Saville talks to Gary Humphreys about the specific challenges faced in developing and distributing SARS-CoV-2 vaccines and the need to fund end-to-end approaches to support those aims.

Q: When did you become interested in virology?

A: At medical school. I wanted to be a paediatric oncologist, but during my first month at university I took a course in molecular biology that got me hooked on viruses. I went on to do a bachelor’s in molecular biology and a master’s in medical virology and after university went into the vaccine industry.

Q: Where did your career start?

A: My first job was with the pharmaceutical company Wyeth where I started out working on a live, attenuated intranasal influenza vaccine. From there I did a lot of research and development relating to seasonal and pandemic flu, but I’ve worked on a number of diseases, including dengue, Ebola, HIV (human immunodeficiency virus) RSV (respiratory syncytial virus) and *E. coli* (*Escherichia coli*).

Q: You left the private sector in 2017. What prompted that move?

A: There was an opportunity to work with the nascent Coalition for Epidemic Preparedness Innovations (CEPI) and I took it. Despite having been very happy working in industry, I joined CEPI in November 2017. Perhaps I’m a glutton for punishment! Seriously though, I was lucky enough to be part of the deliberations that went into the organization’s formation as an industry representative, so I had a good idea of what I was getting into.

Q: What does CEPI do?

A: Essentially CEPI came out of the failure of the market to respond to the Ebola outbreaks of 2014-2015 and the need for research and development into priority pathogens. CEPI exists to support such research and development, and to make sure that vaccines are made available to lower-income countries. We currently have a portfolio of vaccine candidates for five priority diseases, namely Lassa, MERS (Middle East Respiratory syndrome), Nipah, Chikungunya and Rift Valley fever. We also support Ebola vaccine research and development, and of course we are now very focused on vaccine development for SARS-CoV-2.

Q: How many vaccine candidates for SARS-CoV-2 is CEPI supporting?

A: We currently have a portfolio of nine vaccine candidates, relying on four distinct bio-technologies. We have cast the net quite wide because, at this point, we don’t really know which platform is going to be successful or whether multiple platforms may be needed. You need multiple shots on target to improve your chances of scoring a goal.

“You need multiple shots on target to improve your chances of scoring a goal.”

Q: What are your criteria for selection?

A: Part of CEPI’s remit is rapid response, so we focus on vaccines based on technologies that can move forward rapidly and can be scaled. As an example, we fund ground-breaking nucleic acid-based platforms, which permit the development of vaccines to the stage of clinical trials in weeks rather than years. The much-talked-about messenger RNA (mRNA)-based vaccines being developed for SARS-CoV-2 are a case in point.

Q: Can you explain how those vaccines work in simple terms?

A: Essentially, the mRNA vaccines instruct the body to make target proteins – such as the protein spike used by the SARS-CoV-2 virus to gain entry to certain cells – that, when registered by the immune system, elicit the required immune response.

Q: In some cases, regulators are seeking to expedite vaccine development by introducing new flexibilities, for example allowing animal and human trials to be conducted in parallel; does this increase the risk of harming people who participate in trials?

A: It’s true that there is tremendous pressure to move forward quickly. However, it is our position that testing for safety and effectiveness needs to be done, and needs to be done properly, and that includes appropriate pre-clinical testing. That said, I think the jury is out regarding what the right testing models are. The level of testing that will be required before moving into clinical trials will vary from candidate to candidate and will depend on how well-known that candidate vaccine platform is. For example, a candidate vaccine platform that has an established safety profile can be advanced more quickly than a completely novel vaccine. But the minimum safety and efficacy data that would be required for any vaccine is still expected. And there are other ways to move forward quickly, such as manufacturing at risk, but that is a financial risk.

Q: It has been estimated that only 10% of vaccines make it from the laboratory to final approval, while no vaccine against a coronavirus, including MERS, has yet to be licensed. Does this concern you?

A: Of course. However, I believe we can improve our chances of success by doing a very careful review of the candidates under consideration and by assessing which have the profile most likely to succeed. And while it is true there is still no MERS vaccine, some candidates are in clinical trials. Also, we have learnt a great deal from their development and it is not surprising that some of the most advanced SARS-CoV-2 candidate vaccines are coming out of labs that have worked on MERS. The University of Oxford vaccine is a good example. Because of their work on MERS, researchers there had a strong appreciation of how these viruses work and the assays required to test for immune response. That was a great advantage in January when news of SARS-CoV-2 first emerged.

Q: Presuming a vaccine is eventually shown to be safe and effective, what will be the biggest challenges in getting it to the billions of people who need it?

A: Many of the challenges will be familiar, such as ensuring quality in the manufacturing of biological products, with all the demands such manufacturing entails. However particular challenges will apply to the manufacture SARS-CoV-2. Indeed, these challenges already do apply because in order to meet demand for billions of doses in a timely manner we have to start building manufacturing capacity now. That means setting up multiple plants for products that may never be made – manufacturing at risk.

Q: Where should manufacturing facilities be built?

A: CEPI favours scaling up (making plants bigger) and scaling out (setting up plants in multiple locations) to ensure the biggest global manufacturing footprint, and we are currently conducting a survey of local manufacturing capacity for the essential ingredients of the vaccine, and for the things needed to arrive at a final product, such as vials, labelling and packaging. We think the manufacturing facilities exist, but they need to be adapted to the manufacturing of the SARS-CoV-2 vaccines. Of course, manufacturing is only one of the challenges we face. Procurement, allocation and delivery challenges will also have to be overcome in very short order, a feat that will require an end-to-end multi-organizational approach.

“We have to start building manufacturing facilities now.”

Q: What do you mean by an end-to-end approach?

A: An approach that brings together the expertise across the product value chain, from research and development to final allocation and use. Such an approach underpins the ACT Accelerator initiative which was launched at the end of April and brings together key players, including CEPI, Gavi, the Vaccine Alliance and of course the World Health Organization (WHO).

Q: What is CEPI’s role within the ACT Accelerator?

A: CEPI has responsibility for handling research and development and manufacturing under the initiative, while GAVI handles procurement and delivery, and WHO is handling allocation and policy. Working in collaboration with other partners, these organizations are working together so that everyone involved can be fully aware of what is happening upstream and make their preparations accordingly.

Q: According to a recently published WHO investment case, only US$3.4 billion had been pledged for the ACT Accelerator by 26 June. This is US$28 billion short of what is required. Does this concern you?

A: CEPI was already fundraising before the ACT Accelerator and raised US$1.4 billion, US$895 million which we spent in the first half of the year. So, while we still have some funds to invest, we are a little concerned about the overall funding situation, especially given the commitment we made to providing 2 billion doses of vaccine by the end of 2021. If we really want to deliver that volume of vaccine, we have to act now, and that means committing the funds required according to the investment case.

Q: Why has funding for the ACT Accelerator been so lacklustre?

A: One of the challenges we face is that several countries have decided to go their own way, making bilateral deals, where one country commits to buying from a manufacturer in another country. It is perhaps natural, or at least human nature, that each country wants to protect its own population, but in a situation like the one with which we are confronted, bilateral agreements present some important disadvantages. First, they exclude those countries that are not in a position to make bilateral arrangements, leaving much of the global population out in the cold. Those are the countries that CEPI was created to serve, ensuring that they are not always last in line for urgently needed medical products. Second, bilateral accords focusing on one or two vaccines exposes individual countries to the risk of backing the wrong candidates. At this stage we still don’t know which candidates are going to be successful. We have funded a large portfolio of 9 vaccine candidates to date. So, we really need to come together, pooling risks and resources, giving us all a higher probability of success.

Q: Where do you think we will stand a year from now?

A: Presuming we have a viable candidate vaccine a year from now, because of our commitment to manufacturing at risk, we should have sizable quantities of vaccine available. However, there will be a shortage, and that is why it is vital to establish a fair allocation system right now. We need to make sure that those people who need the vaccine get it, because there will be a shortage for some time.

